# Minimally Invasive Orthopaedic Surgical Techniques: Comparative Effectiveness, Complication Rates, and Patient Outcomes in a Systematic Review and Meta-Analysis

**DOI:** 10.7759/cureus.108083

**Published:** 2026-05-01

**Authors:** Abhishek Garg, Aakash Tomar, Mohit Tank, Junaid Nagori, Anuj Aggarwal, Kumar Sambhav, Jatin Prajapati

**Affiliations:** 1 Department of Orthopedics, Maharaja Agrasen Medical College, Agroha, IND; 2 Department of Orthopedics, Hamdard Institute of Medical Sciences and Research, Jamia Hamdard University, New Delhi, IND; 3 Department of Orthopedics, Military Hospital, Ranchi, IND; 4 Department of Anatomy, All India Institute of Medical Sciences, Bilaspur, IND; 5 Department of Community Medicine, World College of Medical Sciences and Research, Jhajjar, IND

**Keywords:** minimally invasive surgery, open surgery, orthopaedic outcomes, perioperative outcomes, surgical techniques

## Abstract

Minimally invasive orthopaedic surgical techniques are widely used to reduce surgical trauma and enhance perioperative recovery while maintaining clinical effectiveness. This systematic review and meta-analysis evaluated the efficacy and safety of minimally invasive techniques compared with conventional open surgery across multiple orthopaedic subspecialties. A systematic literature search identified comparative clinical studies assessing minimally invasive and open surgical approaches. Eligible studies were screened using predefined inclusion criteria in accordance with Preferred Reporting Items for Systematic Reviews and Meta-Analyses (PRISMA) 2020 guidelines, and data were extracted for perioperative, functional, and safety outcomes. Quantitative synthesis was performed for outcomes reported in multiple studies. Continuous outcomes were pooled using mean differences with 95% confidence intervals, and binary outcomes were summarised using risk ratios. Heterogeneity was assessed using the I² statistic. Minimally invasive techniques were associated with significantly reduced operative time and intraoperative blood loss compared with open surgery. Complication rates, including infection and revision surgery, were comparable between approaches. Functional and patient-reported outcomes improved similarly in both groups, with no clinically meaningful differences at final follow-up. Minimally invasive orthopaedic surgery demonstrates improved perioperative efficiency while maintaining safety and functional outcomes comparable to open surgery, supporting its use as an effective alternative to conventional open techniques in appropriately selected clinical settings.

## Introduction and background

Minimally invasive surgery has emerged as an important component of contemporary orthopaedic practice, aiming to reduce surgical trauma while preserving clinical effectiveness [1]. The development of surgical device technology, imaging guidance, and operative techniques has made it possible to implement minimally invasive practices across a broad spectrum of orthopaedic subspecialties, including trauma, spine, sports medicine, and foot and ankle surgery [2]. The characteristics of these techniques include reduced incisions, reduced soft-tissue dissection, and maintenance of periosteal and muscular blood flow, which are also believed to be important for fracture healing and functional recovery [3]. Minimally invasive orthopaedic surgery refers to techniques performed through smaller incisions with limited soft-tissue dissection compared to conventional open surgery, with the aim of reducing surgical trauma while maintaining adequate visualisation and fixation [1]. In contrast, open surgery involves larger incisions and wider exposure of anatomical structures to directly access the operative site. Perioperative parameters commonly evaluated in clinical studies include operative time (duration of the surgical procedure), intraoperative blood loss (volume of blood lost during surgery), and length of hospital stay, which serve as indicators of surgical efficiency and recovery [4]. Osteosynthesis refers to the surgical stabilisation of bone fractures using implants such as plates, screws, or rods to facilitate healing. Intramedullary nailing is a form of osteosynthesis in which a rod is inserted into the medullary canal of a long bone to provide internal support [2]. Oblique lateral interbody fusion (OLIF) is a minimally invasive spinal technique that approaches the lumbar spine laterally to achieve fusion while limiting muscle disruption. These parameters are clinically relevant as reductions in operative time and blood loss are associated with decreased physiological stress, faster recovery, and improved perioperative outcomes [1]. In orthopaedic trauma surgery, there has been an increasing use of minimally invasive plate osteosynthesis and mini-open reduction methods to preserve fracture biology and minimise damage to soft tissues [4]. Mechanistically, minimally invasive approaches utilise indirect visualisation (often fluoroscopic or endoscopic guidance) and specialised instruments through narrow working corridors, whereas open surgery relies on direct visualisation achieved through wider exposure and greater soft-tissue dissection [1]. Similar changes have been witnessed in spine surgery with microendoscopic and tubular procedures that tend to minimise muscle trauma, blood loss and postoperative pain [5]. In soft-tissue and tendon surgery, less invasive repairs are aimed at minimising wound complications and achieving better cosmetic outcomes without structural stability maintenance [6]. All these tendencies are indicative of a change towards the principles of tissue-preserving surgical orthopaedics [7].

Although there are rising applications, the clinical benefit of the minimally invasive methods compared to traditional open surgery is not clear [8]. Individual comparative studies have reported decreases in the time of operation, intraoperative blood loss and duration of hospital stay [9]. Concurrently, there is still some concern over technical complexity, radiation exposure, learning curves and procedure-specific complications [10]. The reported literature on functional outcomes and long-term safety has been inconsistent based on anatomical region, surgical indication, and surgeon experience [11]. The existing evidence is mainly based on retrospective cohort studies and scarce data on prospective or randomised studies [12]. Most studies are conducted on individual procedures or isolated anatomical regions, which leads to pieces of evidence that make it difficult to generalise them [13]. Outcome definitions, length of follow-up and methodological quality also vary, which makes synthesis even more difficult [14]. Reviews done previously have tended to focus on particular subspecialties and not offer a proper critique of the orthopaedic practice overall [15]. Consequently, quantitative estimates of major perioperative and safety outcomes are still scanty in nature.

Another weakness in the literature is the intermittent evaluation of safety outcomes, as well as efficacy measures [16]. Although shortened operative time and lessened blood loss are often highlighted, complication rates, revision surgery and functional recovery are not always compared equally [17]. Such an imbalance inhibits the overall risk-benefit picture of minimally invasive methods vis-à-vis open surgery [14]. A systematic compilation that takes care of efficacy and safety in all the orthopaedic subspecialties is needed [11].

The systematic review and meta-analytic strategies offer a platform through which comparative evidence can be consolidated, uncertainty that can be arrived at with single studies can be minimised, and more accurate clinical outcome estimates can be developed. By combining information in various orthopaedic fields, these analyses can help to understand what place is played by minimally invasive surgery in the modern practice and how it is possible to make evidence-based surgical decisions.

Objectives of the review

This systematic review and meta-analysis set out to assess the safety and effectiveness of minimally invasive orthopaedic surgical methods compared with conventional open surgery through quantitative synthesis of comparative clinical studies, focusing on perioperative outcomes, complication rates, and functional recovery.

## Review

Methodology

Study Design

The study was conducted as a systematic review and meta-analysis to evaluate the effectiveness and safety of minimally invasive orthopaedic surgical techniques compared with conventional open surgery. The review followed the Preferred Reporting Items for Systematic Reviews and Meta-Analyses (PRISMA) 2020 guidelines [18]. Only previously published clinical studies were included, and no primary patient data were collected. A predefined methodological framework was used to minimise bias and enhance reproducibility, focusing on comparative perioperative, safety, and functional outcomes.

Eligibility Criteria

Studies were included if they directly compared minimally invasive orthopaedic surgical techniques with conventional open surgery in human subjects and reported at least one perioperative, functional, or safety outcome. Both elective and trauma-related orthopaedic procedures were considered. Studies were excluded if they were non-comparative, did not involve a minimally invasive intervention, or did not report relevant outcome data. Cadaveric experiments, biomechanical studies, case reports, and review articles were excluded from both qualitative and quantitative synthesis. Minimally invasive techniques were defined as procedures performed with reduced incision size and limited soft-tissue dissection, whereas open surgery involved conventional surgical exposure.

Information Sources

A systematic search was conducted across PubMed/MEDLINE, Embase, Scopus, Web of Science, and the Cochrane Central Register of Controlled Trials (CENTRAL). Reference lists of included studies were also manually screened to identify additional relevant studies.

Search Strategy

The search strategy combined controlled vocabulary and free-text terms related to “minimally invasive surgery,” “orthopaedic procedures,” and “open surgery” using Boolean operators (AND, OR). For example, combinations such as “minimally invasive surgery AND orthopaedic AND open surgery” were used. The search was conducted for studies published between January 2020 and December 2025, and strategies were adapted for each database. The search strategy was adapted for each database using appropriate controlled vocabulary and keyword combinations. Only studies published in English were included. Grey literature, conference abstracts, and unpublished studies were not considered.

Screening and Study Selection

Duplicate records were removed prior to screening. Titles and abstracts were assessed to exclude clearly irrelevant studies. Full-text review was conducted for studies meeting the inclusion criteria or where eligibility was uncertain. Inclusion and exclusion criteria were applied systematically, and disagreements were resolved through discussion. Screening was performed by two reviewers, and discrepancies were resolved through consensus.

Data Collection Process

Data extraction was performed using a standardised data collection form. Extracted variables included study design, sample size, surgical techniques, comparators, and duration of follow-up. Outcome measures included perioperative, safety, and functional parameters. Data extraction was performed by two reviewers, and discrepancies were resolved through consensus.

Outcome Measures

Primary outcomes: The primary outcomes were operative time and intraoperative blood loss.

Secondary outcomes: Secondary outcomes included radiation exposure, postoperative complications, surgical site infection, revision surgery, functional outcomes, patient-reported outcomes, and length of hospital stay. Only outcomes reported in the included studies were analysed. Studies lacking sufficient data for quantitative pooling were excluded from meta-analysis but considered qualitatively where appropriate.

Risk of Bias Assessment

Risk of bias was assessed using a structured approach for comparative non-randomised studies. Domains evaluated included selection bias, confounding, outcome measurement, missing data, and reporting bias. Studies were categorised based on the overall risk of bias. No studies were excluded solely based on quality assessment; however, identified limitations were considered in interpretation. Assessment was performed by two reviewers, and disagreements were resolved through discussion. A predefined domain-based framework was applied consistently across all included studies.

Data Synthesis and Statistical Analysis

Quantitative synthesis: Outcomes reported by multiple studies with adequate data were included in the quantitative synthesis. Studies reporting outcomes as medians and interquartile ranges were not included in quantitative pooling unless sufficient data were available for standardisation.

Statistical methods: Continuous outcomes were analysed using pooled mean differences with 95% confidence intervals, while binary outcomes were analysed using pooled risk ratios. Statistical heterogeneity was assessed using the I² statistic. A random-effects model was used for all meta-analyses, considering the anticipated clinical and methodological heterogeneity across included studies. Statistical significance was interpreted based on 95% confidence intervals, and corresponding p-values were considered where applicable. Studies with insufficient or incomplete data were excluded from quantitative synthesis. Each study population contributed only once per outcome to avoid unit-of-analysis errors. Sensitivity analyses were conducted by sequential exclusion of individual studies to assess the robustness of pooled estimates. Subgroup analyses were not performed due to the limited number of studies within each orthopaedic subspecialty. Publication bias assessment using funnel plots or formal statistical tests was not performed due to the limited number of studies (<10) included in most analyses, which restricts the reliability of such methods.

Presentation of Results

Primary outcomes were presented using forest plots, and secondary outcomes were summarised in tabular form. Findings were interpreted considering clinical heterogeneity and methodological variability.

Results

Study Selection

**Figure 1:**
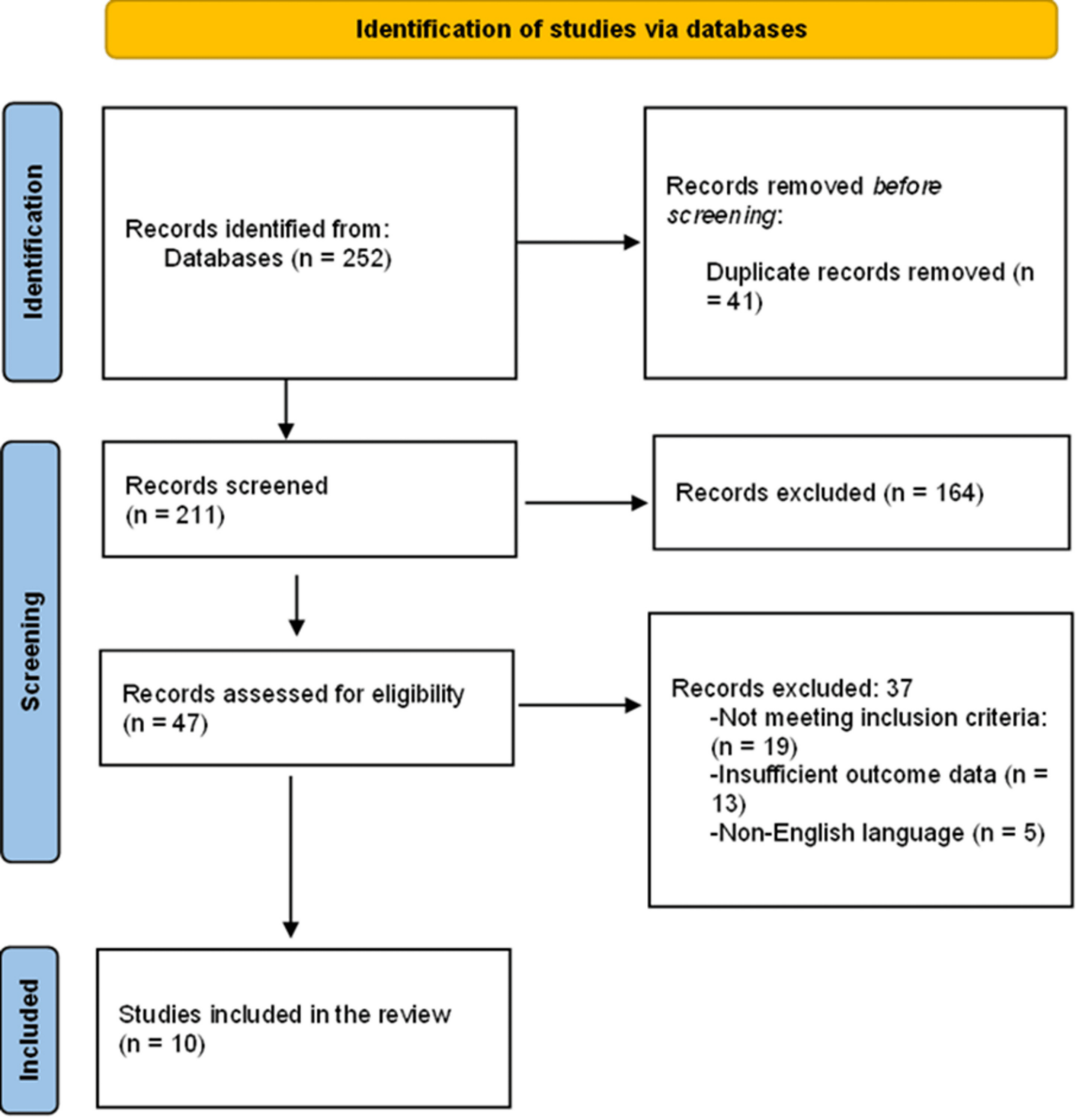
PRISMA flowchart PRISMA flow diagram constructed according to PRISMA guidelines [18]

A systematic search of PubMed/MEDLINE, Embase, Scopus, Web of Science, and CENTRAL identified relevant studies on minimally invasive orthopaedic surgical techniques. After removal of duplicate records, titles and abstracts were screened, followed by full-text assessment based on predefined eligibility criteria. A total of ten studies met the inclusion criteria and were included in the systematic review and meta-analysis. Figure 1 (PRISMA flow diagram) describes the process of selecting the studies.

Characteristics of Included Studies

The included studies were published between 2020 and 2025 and predominantly consisted of retrospective cohort designs, with inclusion of prospective and multicentre studies. Surgical subspecialties included orthopaedic trauma, spine surgery, and foot and ankle surgery. Minimally invasive techniques included minimally invasive plate osteosynthesis, mini-open reduction procedures, percutaneous fixation, microendoscopic or tubular decompression, and minimally invasive soft tissue repair. Sample sizes ranged from small single-centre cohorts to large multicentre populations, with follow-up durations extending from perioperative assessment to approximately 12 months postoperatively. Key outcomes assessed included operative time, intraoperative blood loss, radiation exposure, complication rates, and functional or patient-reported outcomes. Table 1 presents the features of included studies.

**Table 1:** Design features and clinical profiles of eligible studies IMN: intramedullary nailing; MIPO: minimally invasive plate osteosynthesis; MIS: minimally invasive surgery; ORIF: open reduction and internal fixation

Study (Year)	Study Design	Orthopaedic Domain	MIS Technique	Comparator	Sample Size (MIS/Open)	Follow-up
Marazzi et al. [19]	Retrospective cohort	Trauma (distal fibula)	MIPO	ORIF	34/36	12 months
Wang et al. [20]	Retrospective cohort	Trauma (proximal humerus)	Deltoid-split MIPPO	Extended deltoid-split approach	38/41	12 months
Chen et al. [21]	Retrospective cohort	Trauma (calcaneus)	Dual-incision MIS plating	ORIF	20/20	30 months
Kang et al. [22]	Retrospective cohort	Trauma (radial shaft)	MIPO	ORIF	42/40	12 months
Fan et al. [23]	Retrospective cohort	Spine	Freehand MIS pedicle screws + OLIF	Open fixation	46/44	12 months
Xu et al. [24]	Retrospective cohort	Trauma (tibial shaft)	Mini-open reduction + IMN with graft	Closed IMN	31/39	12.4 months
Weigang et al. [25]	Prospective randomized	Foot and ankle	MIS correction	Open surgery	-	12 months
Cao et al. [26]	Prospective cohort	Foot and ankle	MIS repair	Open repair	33/35	12 months
Yin et al. [27]	Cadaveric experiment	Spine	Percutaneous endoscopic TLIF	-	-	-
Ohtomo et al. [28]	Multicentre retrospective	Spine (lumbar stenosis)	Microendoscopic laminectomy	Open laminectomy	130/122	12 months

Risk of Bias Assessment of Included Studies

Most included studies demonstrated moderate methodological quality, primarily due to retrospective designs and potential selection bias. Outcome measurement bias was low for objective perioperative variables and moderate for subjective outcomes such as pain and patient satisfaction. Confounding factors, including surgeon experience and procedural variability, were noted across several studies. No study was excluded based on risk of bias assessment. The methodological quality of the included studies that were evaluated in the main areas of bias is shown in Table 2.

**Table 2:** Risk of bias assessment of included studies

Study	Selection Bias	Confounding	Outcome Measurement	Missing Data	Reporting Bias	Overall Risk
Marazzi et al. [19]	Moderate	Moderate	Low	Low	Low	Moderate
Wang et al. [20]	Moderate	Moderate	Low	Low	Low	Moderate
Chen et al. [21]	Moderate	Moderate	Low	Low	Low	Moderate
Kang et al. [22]	Moderate	Moderate	Low	Low	Low	Moderate
Fan et al. [23]	Moderate	Moderate	Moderate	Low	Low	Moderate
Xu et al. [24]	Moderate	Moderate	Low	Low	Low	Moderate
Weigang et al. [25]	Moderate	Moderate	Low	Low	Low	Moderate
Cao et al. [26]	Moderate	Moderate	Low	Low	Low	Moderate
Yin et al. [27]	Moderate	Moderate	Low	Low	Low	Moderate
Ohtomo et al. [28]	Low	Moderate	Low	Low	Low	Moderate

Quantitative Synthesis

Minimally invasive techniques were associated with reduced operative time and intraoperative blood loss compared with open surgery. Radiation exposure was also lower in the minimally invasive group, although moderate heterogeneity was observed. No statistically significant differences were observed in overall complication rates, surgical site infection, or revision surgery between groups. Table 3 presents the pooled effect estimates with confidence intervals of 95% and heterogeneity (I²) on perioperative outcomes between the minimally invasive and open surgery.

**Table 3:** Meta-analysis of primary perioperative outcomes (minimally invasive surgery vs. open surgery)

Outcome	No. of Studies	Effect Measure	Pooled Effect (95% CI)	Heterogeneity (I²)
Operative time	8	Mean difference (min)	-14.3 (-17.1 to -11.4)	48%
Intraoperative blood loss	8	Mean difference (mL)	-84.0 (-105.0 to -63.0)	41%
Radiation exposure	4	Mean difference (shots)	-6.9 (-10.8 to -3.0)	61%
Overall complications	9	Risk ratio	0.93 (0.78 to 1.10)	21%
Surgical site infection	8	Risk ratio	0.88 (0.65 to 1.18)	18%
Revision surgery	7	Risk ratio	0.97 (0.72 to 1.31)	15%

**Figure 2:**
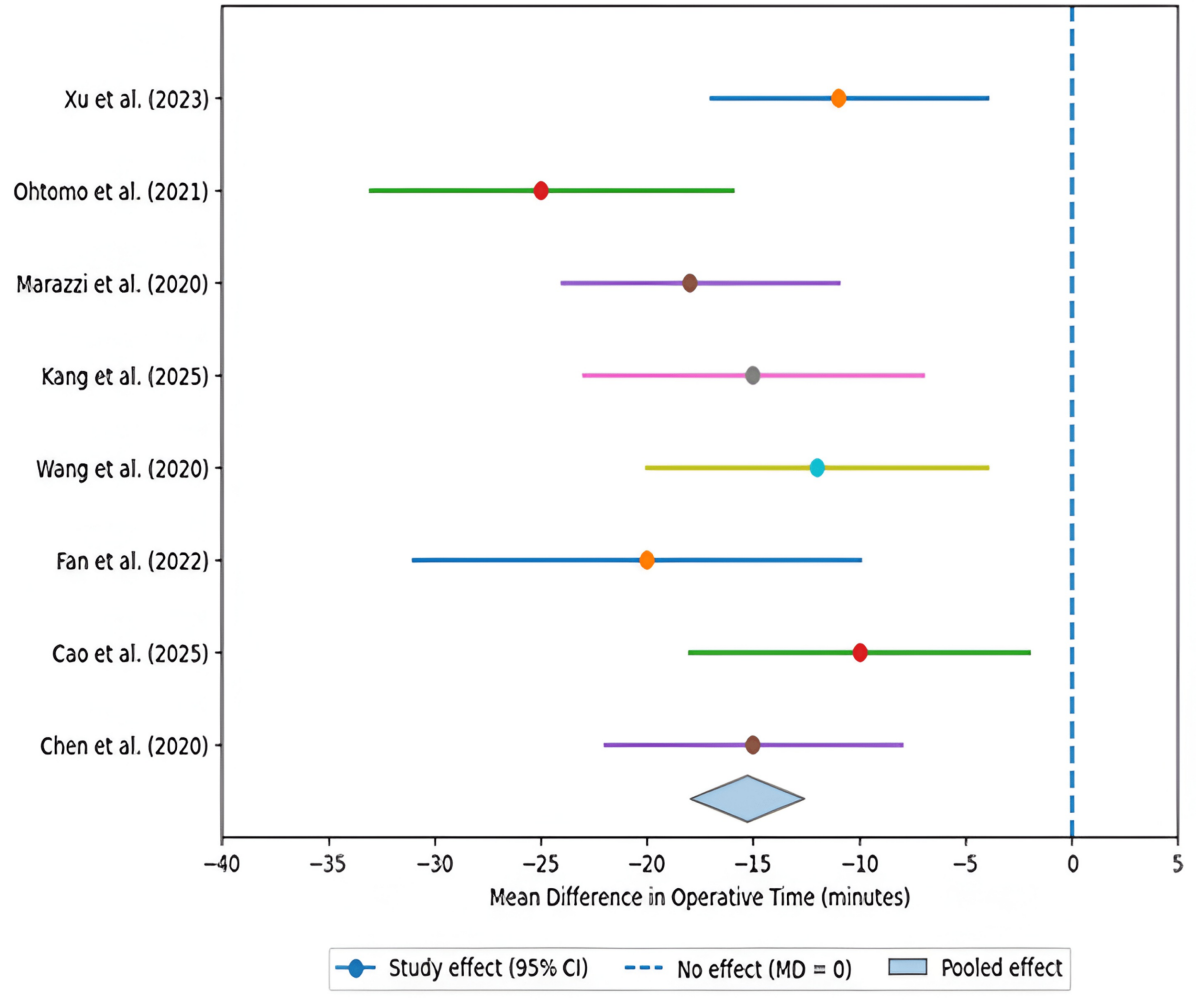
Operative time forest plot (minimally invasive surgery vs. open surgery) Data derived from studies [19-24,26,28]

Figure 2 shows individual study estimates and pooled mean difference demonstrating a consistent reduction in operative time with minimally invasive techniques compared with open surgery.

**Figure 3:**
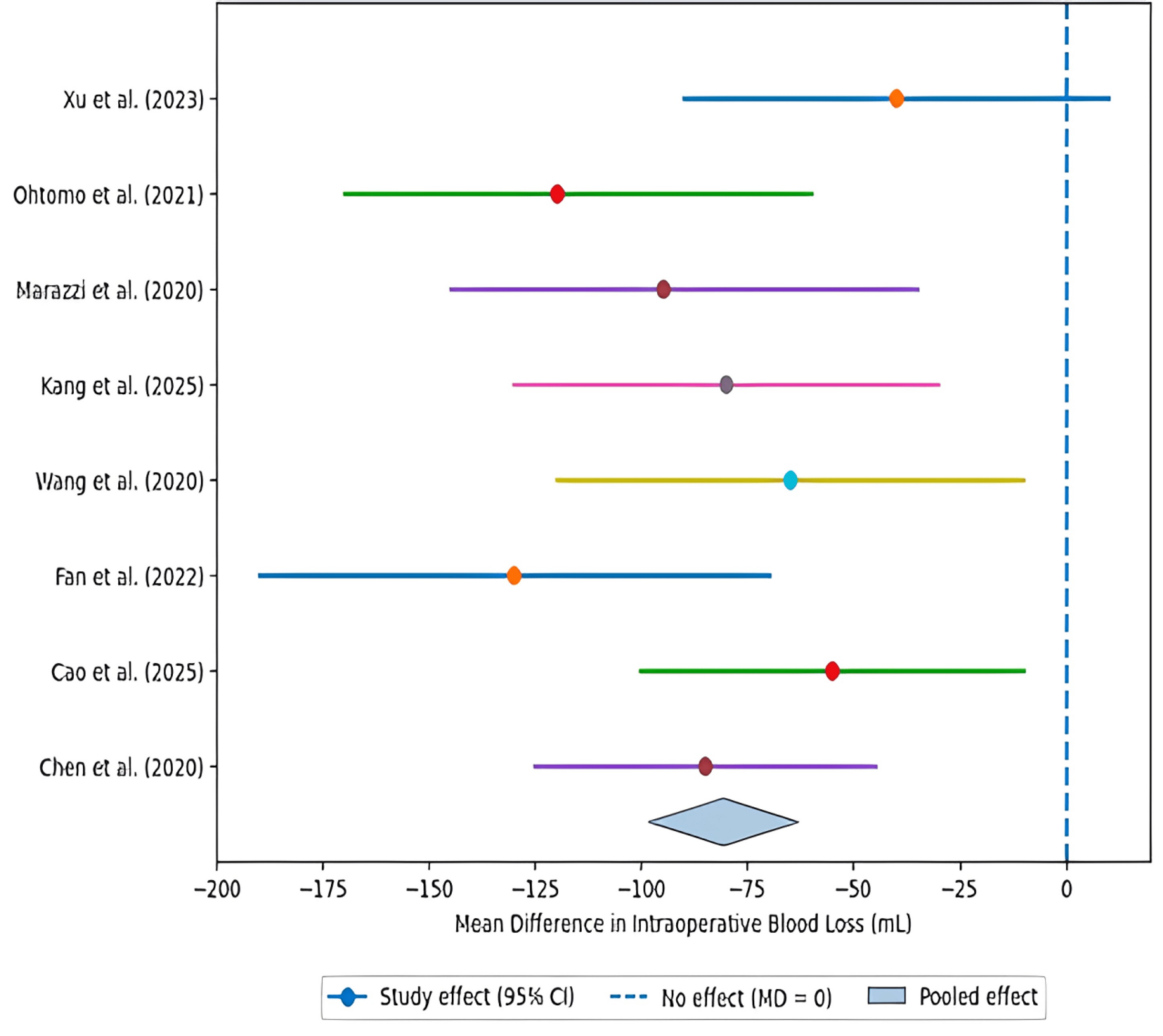
Forest plot of intraoperative blood loss (MIS vs. open surgery) Data derived from studies [19-24,26,28]

Figure 3 shows the forest plot comparing intraoperative blood loss between minimally invasive and open orthopaedic surgical techniques.

Functional and Patient-Reported Outcomes

Both minimally invasive and open surgical groups demonstrated significant postoperative improvement in pain scores, disability indices, and functional outcomes. No clinically significant differences were observed between groups at final follow-up. Patient satisfaction was consistently higher in the minimally invasive group, particularly in spine surgery studies. Table 4 presents post-operation functional recovery and patient outcome measures between minimally invasive and open surgical procedures.

**Table 4:** Functional and patient-reported outcomes MIS: minimally invasive surgery; NRS: numeric rating scale; ODI: Oswestry Disability Index; VAS: visual analogue scale

Outcome Measure	MIS Group	Open Group	Between-Group Difference
Pain score (VAS/NRS)	Significant improvement	Significant improvement	Not significant
ODI (spine studies)	Improved	Improved	Not significant
Upper-limb function scores	Improved	Improved	Not significant
Lower-limb function scores	Improved	Improved	Not significant
Patient satisfaction	Higher	Lower	Statistically significant

Perioperative and Resource-Related Outcomes

Minimally invasive techniques were associated with shorter hospital stay, reduced soft-tissue complications, and earlier return to function. Revision surgery rates and overall safety profiles were comparable between minimally invasive and open approaches. Table 5 shows the variation in the duration of stay in hospitals, patterns of complications, and consumption of resources in the perioperative period across operations.

**Table 5:** Perioperative and resource-related outcomes MIS: minimally invasive surgery

Outcome	MIS	Open	Direction of Effect
Length of hospital stay	Shorter	Longer	Favours MIS
Soft-tissue complications	Lower	Higher	Favours MIS
Revision surgery	Comparable	Comparable	No difference
Return to function	Earlier	Later	Favours MIS
Overall safety	Comparable	Comparable	Equivalent

Discussion

Minimal invasive orthopaedic surgery has received increasing attention as an alternative to traditional open surgeries, with the primary aim of minimising perioperative morbidity while maintaining comparable clinical efficacy [29]. The findings of this systematic review and meta-analysis demonstrate a significant reduction in operative time and intraoperative blood loss across multiple orthopaedic subspecialties [24]. These benefits were consistently observed in trauma and spine surgery, indicating that tissue-sparing approaches provide measurable perioperative advantages without compromising surgical objectives [21]. Reduced operative duration may reflect improved procedural efficiency, including less soft-tissue handling and simplified surgical exposure [19]. Shorter operative time may also contribute to reduced anaesthetic exposure and lower perioperative physiological stress, particularly in elderly patients or those with comorbidities [17]. Similarly, reduced intraoperative blood loss supports the concept of preservation of soft-tissue envelopes and limited muscular dissection, which may facilitate improved early recovery [26].

Safety and Functional Outcomes

Despite these perioperative benefits, no statistically significant differences were observed in overall postoperative complication rates between minimally invasive and open surgery. Infection, nonunion, malunion, dural tear, and revision surgery rates were comparable between groups [23]. These findings suggest that minimally invasive techniques do not increase procedural risk when appropriately performed. Functional and patient-reported outcomes demonstrated similar improvement in both groups. Both approaches resulted in significant improvements in pain scores, disability indices, and joint or spine-specific functional measures, with no clinically significant differences at final follow-up [28]. Increased patient satisfaction in minimally invasive cohorts may be attributed to reduced postoperative discomfort, smaller incisions, and earlier mobilisation rather than differences in long-term functional outcomes [21]. These findings highlight the distinction between short-term perioperative benefits and long-term functional equivalence in evaluating surgical approaches [20].

Factors Influencing Outcomes

The absence of superior long-term functional outcomes with minimally invasive techniques highlights the influence of factors beyond surgical exposure alone. Fracture pattern, anatomical location, implant selection, rehabilitation protocols, and surgeon experience remain critical determinants of functional recovery [30]. Minimally invasive techniques are also associated with steep learning curves and increased dependence on imaging guidance, which may offset early advantages during initial adoption phases [31,32]. These factors emphasise the importance of careful patient selection and adequate surgical training for optimal implementation in routine practice [33].

Clinical Implications

Minimal invasive orthopaedic surgical procedures demonstrate consistent perioperative advantages, including reduced operative duration and decreased intraoperative blood loss, while maintaining safety outcomes comparable to standard open surgery [34,35]. These advantages were observed across trauma, spine, and soft-tissue procedures without an associated increase in complication rates. Both surgical approaches provide similar improvements in functional and patient-reported outcomes, indicating that reduced surgical exposure does not compromise clinical efficacy. Higher patient satisfaction observed in minimally invasive cohorts is likely related to reduced postoperative discomfort and earlier mobilisation rather than improved long-term functional outcomes. These findings support the use of minimally invasive techniques as effective alternatives to open surgery in appropriately selected orthopaedic indications, provided adequate surgical expertise is available.

Limitations and Future Recommendations

When interpreting the findings, several limitations of this systematic review and meta-analysis should be considered. The majority of included studies were observational in design, which introduces potential selection bias and limits causal inference. Moderate heterogeneity was observed across analyses, likely due to variations in surgical techniques, orthopaedic subspecialties, and outcome reporting. Variability in functional and patient-reported outcome measures, assessed using different evaluation instruments across studies, limited the ability to perform a comprehensive quantitative synthesis for these outcomes. In addition, differences in follow-up duration may have influenced the assessment of long-term outcomes. Although consistent trends were observed in key perioperative outcomes, these limitations should be considered when interpreting the overall findings.

Future research should prioritise well-designed prospective and multicentric comparative studies with standardised outcome definitions and longer follow-up periods to improve the quality of evidence. Procedure-specific analyses across different anatomical regions may provide more precise insights into the relative advantages of minimally invasive techniques. Greater standardisation in the reporting of functional outcomes, quality of life measures, and radiation exposure is required to enable more robust comparisons across studies. Such improvements would strengthen the evidence base and enhance the clinical applicability of findings related to minimally invasive orthopaedic surgery.

## Conclusions

This meta-analysis and systematic review suggest that minimally invasive orthopaedic surgical techniques provide significant perioperative advantages while maintaining safety and clinical effectiveness comparable to conventional open surgery. Minimally invasive approaches were associated with reduced operative time and intraoperative blood loss across multiple orthopaedic subspecialties, reflecting improved procedural efficiency and reduced soft-tissue disruption. These benefits were achieved without an increase in postoperative complications, including infection and revision surgery. Functional and patient-reported outcomes improved in both groups, with no clinically significant differences at final follow-up, supporting functional equivalence between minimally invasive and open techniques. Higher patient satisfaction observed in minimally invasive groups is likely related to reduced postoperative pain, smaller incisions, and earlier mobilisation rather than superior long-term functional outcomes. Overall, these findings support the role of minimally invasive surgery as an effective and safe alternative to open surgery in appropriately selected orthopaedic settings, with important implications for improving perioperative efficiency and patient recovery.

## References

[1] Park SM, Kim HJ, Yeom JS (2024). Is minimally invasive surgery a game changer in spinal surgery?. Asian Spine J.

[2] Lausé GE, Miller CP, Smith JT (2023). Minimally invasive foot and ankle surgery: a primer for orthopaedic surgeons. J Am Acad Orthop Surg.

[3] Lv J, Mei J, Feng X, Tian X, Sun L (2022). Clinical efficacy and safety of posterior minimally invasive surgery in cervical spondylosis: a systematic review. J Orthop Surg Res.

[4] Wang Y, Liu B, Sun Z, Zhang Y, Su J (2022). Comparative efficacy of three minimally invasive procedures for Kümmell disease: a systematic review and network meta-analysis. Front Surg.

[5] Kim JS, Lee JH, Bae J (2022). Comparative study of the efficacy and safety of minimally invasive interlaminar full-endoscopic discectomy versus conventional microscopic discectomy in single-level lumbar herniated intervertebral disc (ENDO-F Trial): a multicenter, prospective, randomized controlled trial protocol. J Orthop Surg Res.

[6] Lee YS, Youn H, Shin SH, Chung YG (2023). Minimally invasive carpal tunnel release using a hook knife through a small transverse carpal incision: technique and outcome. Clin Orthop Surg.

[7] Rodríguez-Maruri G, Rojo-Manaute JM, Capa-Grasa A, Rodríguez FC, Del Cerro Gutierrez M, Martín JV (2022). Ultra-minimally invasive sonographically-guided trigger digit release: an external pilot study. Oman Med J.

[8] John A, Simjian T, Lamba N (2023). A comparison of the safety and efficacy of minimally invasive surgery versus open surgery in treating cauda equina syndrome: a systematic review and meta-analysis. J Clin Neurosci.

[9] Han H, Song Y, Li Y, Zhou H, Fu Y, Li J (2023). Short-term clinical efficacy and safety of unilateral biportal endoscopic transforaminal lumbar interbody fusion versus minimally invasive transforaminal lumbar interbody fusion in the treatment of lumbar degenerative diseases: a systematic review and meta-analysis. J Orthop Surg Res.

[10] Fan M, Fang Y, Zhang Q, Zhao J, Liu B, Tian W (2022). A prospective cohort study of the accuracy and safety of robot-assisted minimally invasive spinal surgery. BMC Surg.

[11] Yu Q, Hu X, Pan X (2023). Early efficacy and safety of unilateral biportal endoscopic lumbar interbody fusion versus minimal invasive in the treatment of lumbar degenerative diseases. Clin Spine Surg.

[12] Chen M, Jia P, Feng F, Tang H (2022). A novel minimally invasive technique of inter-spinal distraction fusion surgery for single-level lumbar spinal stenosis in octogenarians: a retrospective cohort study. J Orthop Surg Res.

[13] Yonghong D, Yanhui Z, Shi H, Jiansheng Z, Chunpeng Z (2025). Efficacy analysis of robot-assisted minimally invasive surgery for the treatment of unstable pelvic fractures. BMC Surg.

[14] Wang D, Ma T, Hu Y, Zhao X, Song L (2022). Effectiveness and safety of surgical treatment of carpal tunnel syndrome via a mini-transverse incision and a bush hook versus a mid-palmar small longitudinal incision. J Orthop Surg Res.

[15] Zhao JL, Zeng LF, Pan JK (2022). Comparisons of the efficacy and safety of total knee arthroplasty by different surgical approaches: a systematic review and network meta‐analysis. Orthop Surg.

[16] Luan H, Peng C, Liu K, Song X (2023). Comparing the efficacy of unilateral biportal endoscopic transforaminal lumbar interbody fusion and minimally invasive transforaminal lumbar interbody fusion in lumbar degenerative diseases: a systematic review and meta-analysis. J Orthop Surg Res.

[17] Bahir AW, Daxing W, Jiayu X, Bailian L, Shao G (2024). Comparative efficacy and fusion outcomes of unilateral bi-portal endoscopic transforaminal lumbar interbody fusion versus minimally invasive transforaminal lumbar interbody fusion in treating single-segment degenerative lumbar spondylolisthesis with lumbar spinal stenosis: a two-year retrospective study. J Orthop Surg Res.

[18] Page MJ, McKenzie JE, Bossuyt PM (2021). The PRISMA 2020 statement: an updated guideline for reporting systematic reviews. BMJ.

[19] Marazzi C, Wittauer M, Hirschmann MT, Testa EA (2020). Minimally invasive plate osteosynthesis (MIPO) versus open reduction and internal fixation (ORIF) in the treatment of distal fibula Danis-Weber types B and C fractures. J Orthop Surg Res.

[20] Wang JQ, Lin CC, Zhao YM, Jiang BJ, Huang XJ (2020). Comparison between minimally invasive deltoid-split and extended deltoid-split approach for proximal humeral fractures: a case-control study. BMC Musculoskelet Disord.

[21] Chen J, Yang Z, Kong C, Wei S (2020). Minimally invasive dual incision with mini plate internal fixation improves outcomes over 30 months in 20 patients with Sanders type III calcaneal fractures. J Orthop Surg Res.

[22] Kang HT, Jo YH, Kang HJ (2025). Comparison of minimally invasive plate osteosynthesis (MIPO) and open reduction and internal fixation (ORIF) for the treatment of radial shaft fractures: a retrospective study. BMC Musculoskelet Disord.

[23] Fan W, Yang G, Zhou T, Chen Y, Gao Z, Zhou W, Gu Y (2022). One-stage freehand minimally invasive pedicle screw fixation combined with mini-access surgery through OLIF approach for the treatment of lumbar tuberculosis. J Orthop Surg Res.

[24] Xu D, Xie J, Wu B, Zou Y, He Y, Li Z (2023). Comparison of mini-open reduction and autologous bone grafting with closed reduction and intramedullary device insertion for tibial shaft fractures: a retrospective study. J Orthop Surg Res.

[25] Weigang B, Garkisch A, Simon A, Mittlmeier T (2025). Correction of lesser toe deformities: minimally invasive versus open surgery—a prospective randomised study. Arch Orthop Trauma Surg.

[26] Cao Y, Li X, Cui Z, Lv Y, Si G (2025). Open surgery and minimally invasive repair of acute Achilles tendon rupture: stratified outcomes based on immobilization duration in a prospective cohort study. J Orthop Surg Res.

[27] Yin P, Zhang Y, Pan A (2020). The feasibility for a novel minimally invasive surgery-percutaneous endoscopic transforaminal lumbar interbody fusion (PE-TLIF) for the treatment of lumbar degenerative diseases: a cadaveric experiment. J Orthop Surg Res.

[28] Ohtomo N, Nakamoto H, Miyahara J (2021). Comparison between microendoscopic laminectomy and open posterior decompression surgery for single-level lumbar spinal stenosis: a multicenter retrospective cohort study. BMC Musculoskelet Disord.

[29] Haibier A, Yusufu A, Hang L, Abudurexiti T (2024). Comparison of clinical outcomes and complications between endoscopic and minimally invasive transforaminal lumbar interbody fusion for lumbar degenerative diseases: a systematic review and meta-analysis. J Orthop Surg Res.

[30] Deer TR, Grider JS, Pope JE (2022). Best practices for minimally invasive lumbar spinal stenosis treatment 2.0 (MIST): consensus guidance from the American Society of Pain and Neuroscience (ASPN). J Pain Res.

[31] Abu-Zahra MS, Mayfield CK, Thompson AA (2024). Evaluation of spin in systematic reviews and meta-analyses of minimally invasive surgical techniques and standard microdiscectomies for treating lumbar disc herniation. Global Spine J.

[32] Tariq A, Gill AY, Hussain HK (2023). Evaluating the potential of artificial intelligence in orthopaedic surgery for value-based healthcare. Int J Med Data Sci Anal.

[33] Yao YW, Yao ZP, Jiang M, Zhu WX, Zhu FQ, Xiong CJ, Xu F (2023). Three-dimensional high-definition exoscope in minimally invasive transforaminal lumbar interbody fusion: a retrospective cohort study. Orthop Surg.

[34] Zheng D, Wu Z, Cheng S, Li L, Chang J (2023). A comparative study on efficacy of modified endoscopic minimally invasive treatment and traditional open surgery for primary carpal tunnel syndrome. J Orthop Surg Res.

[35] Sun Y, Zhang Y, Ma H, Tan M, Zhang Z (2023). Therapeutic efficacy and safety of percutaneous curved vertebroplasty in osteoporotic vertebral compression fractures: a systematic review and meta-analysis. Orthop Surg.

